# Isolation and production optimization of a novel milk-clotting enzyme *Bacillus velezensis* DB219

**DOI:** 10.1186/s13568-022-01493-9

**Published:** 2022-11-26

**Authors:** Yao Zhang, Jiayun Hu, Xiaofeng Liu, Chunmin Jiang, Juan Sun, Xinjie Song, Yuanfeng Wu

**Affiliations:** 1 School of Biological and Chemical Engineering, Zhejiang University of Science and Technology, 318 Liuhe Road, Hangzhou, 310023 Zhejiang China; 2 College of Food Science and Technology, Zhejiang University of Technology, Hangzhou, 310014 Zhejiang China

**Keywords:** Milk-clotting enzyme, Isolation, *Bacillus velezensis* DB219, Fermentation optimization, Response surface methodology

## Abstract

The milk-clotting enzyme (MCE) is a crucial ingredient in cheese manufacture. Due to the limits of traditional MCE, finding viable substitute is a pressing issue. This study aims to isolate and identify a wild strain with high milk-clotting activity (MCA) and low proteolytic activity (PA) and optimize the fermentation conditions for MCE production. A strain of *Bacillus velezensis* DB219 with high MCA/PA value (9.2) was isolated from dairy soil (Wuchang, Heilongjiang, China) and identified through 16S rRNA from 40 strains. The optimal wheat bran, carbon, nitrogen, inoculum size, volume and initial pH were 60 g/L, soluble starch 12.5 g/L, corn steep liquor 3 g/L, 5%, 40 mL and 6.15, respectively for improving DB219 MCE production through single factor experiment. The wheat bran concentration, corn steep liquor concentration and volume were the most critical factor and their changed range was determined through Plackett–Burman design and the steepest ascent/descent experiments. The response surface analysis experiment of three factors and three levels was conducted by Box–Behnken design. The theoretical optimal fermentation conditions for DB219 MCE were as follows: wheat bran concentration 60.14 g/L, soluble starch 12.5 g/L, corn steep liquor 3 g/L, inoculum size 5%, volume 40.08 mL and initial pH 6.15. DB219 MCE achieved the maximal MCA (3164.84 SU/mL) that was 101.9% of the predicted value (3104.49 SU/mL) and 4.3-fold higher than the control.

**Supplementary Information:**

The online version contains supplementary material available at 10.1186/s13568-022-01493-9.

## Introduction

Milk-clotting enzyme (MCE) can make milk coagulation and play a major part in cheese production and dairy processing. The MCE influences cheese yield, texture and flavor. The traditional MCE (e.g., calf rennet, lamb MCE and camel MCE) was the most widely used in the past years (Zhang et al. 2013). However, it is limited in humanistic morality, growth cycle and increased cheese consumption. It attracts great interest in finding traditional MCE substitutes. The milk-clotting activity (MCA) to proteolytic activity (PA) ratio is an inherent characteristic of MCE. The ideal traditional MCE substitute is characterized with a high MCA and a low non-specific PA (Meng et al. 2018; Liu et al. 2021). The high MCA/PA ratio leads to less bitterness and better cheese texture and sense properties. Therefore, MCA/PA ratio is a critical factor in evaluating the applicability of traditional MCE substitute (Ben et al. 2017).

The traditional MCE substitutes can be mainly obtained from plant and microorganism. Plant-derived MCE is mainly produced from root, stem, leave, petal, fruit and other parts of the plants (Gutiérrez-Méndez et al. 2019). However, most plant-derived MCE exhibited low MCA/PA ratio, poor cheese yield and bitter substances in cheese making (Salehi et al. 2017). Recently, microbial MCE attracted much attention as traditional MCE substitutes. Partly fungal MCE from *Rhizomucor miehei* is used in commercial cheese production. Most fungal MCE are strong thermostability and cause high residual PA and bitterness (Celebi et al. 2016). In comparison with fungus, bacteria is more potential due to greater biochemical diversity, easier fermentation control, higher material utilization ratio and easier genetic modification (Luo et al. 2018; Li et al. 2019). Some bacterial MCE showed high MCA/PA ratio (Zhang et al., 2013). However, bacterial MCE are less frequently studied than fungal MCE. Bacterial MCE as traditional MCE substitutes used in food industry are few reported. Therefore, it is necessary to find various MCE-produced bacteria for traditional MCE substitute development.

Wuchang (Heilongjiang, China) is famous for the Wuchang rice. It is known as one of the most famous “black land” in China and rich in nutritional ingredient in agricultural soil. Therefore, this “black land” provides great nutrition environment for the growth and enrichment of microbial species. Some proteases (e.g., serine and threonine protein kinase) produced from microorganism have previously been isolated from the “black land” soil. In this study, a strain of *Bacillus velezensis* DB219 with high MCA/PA value was screened from the “black land”. *Bacillus velezensis* is known for biological control and plant growth promotion. However, few researches on its application in pharmaceutical and food industries have been reported (Adeniji et al. 2019; Zhao et al. 2021). In order to increase the DB219 MCE production, the fermentation conditions were optimized by single factor and response surface methodology.

## Materials and methods

### Chemicals and reagents

Skim milk powder were purchased from Sigma-Aldrich (St. Louis, Missouri, USA). Primary screening medium was solid casein medium (pH 6.8) containing casein peptone 2.5 g/L, skim milk powder 25 g/L, casein 10 g/L, glucose 10 g/L, yeast extract powder 1 g/L and agar 20 g/L. Seed liquid medium was modified TYC medium containing casein peptone 15 g/L, Na_2_HPO_4_·12H_2_O 2 g/L, NaCl 1 g/L, NaHCO_3_ 2 g/L, L-cystine 0.2 g/L, glucose 50 g/L and yeast paste 5 g/L. The fermentation medium was wheat bran shorts (30 g/L) containing carbon 39.28% (w/w), nitrogen 3.12% (w/w) and the moisture 6.51% (w/w). Wheat bran was purchased from Qingdao Yutafeng Trading Co. Ltd. (Shandong, China). The bacterial genomic DNA extraction kit, rTap and related primers were purchased from Takara Biomedical Technology Co., Ltd. (Beijing, China). The other chemicals used in the study were analytical grade from Sinopharm Chemical Reagent Co., Ltd. (Shanghai, China).

### Screening and isolation of MCE-producing strain

The bacterial strains were isolated from soil samples taken from different local regions including sports fields, barns, milking places, drinking areas and producing areas in the open pastures in Heilongjiang. Top soil samples (10–15 cm) were collected, filtered through a 3–4 mm mesh screen and kept at 4 °C. Suspending 1 g of soil sample in 9 mL of sterile saline to create a soil suspension. Executing tenfold serial dilutions to get a final dilution of 10^–4^. A 120 μL aliquot of each diluted sample was cultured in the solid casein medium at 37 °C for 36 h. Each strain was assigned a unique identification number and the width of hydrolysis and precipitation rings were measured every 12 h. The colony that formed a hydrolysis ring and a precipitation ring was regarded as a potential strain and used for further study.

### Identification and characterization of strain

On a modified TYC medium, prospective MCE-producing strains were cultivated and examined utilizing physiological and biochemical characteristics testing. Strain cultures were also evaluated using Bergey’s manual of systematic bacteriology for starch hydrolysis (Xu et al. 2021), Voges-Proskauer reaction (VP), methyl red reaction (MR), hydrogen sulfide gas production, citrate utilization, glucose oxidative fermentation, catalase reaction according to standard protocols (Alippi et al. 2019).

The isolates (n = 15) with high ratios of (i) precipitation ring diameter to hydrolysis ring diameter and (ii) precipitation ring diameter to colony zone diameter were chosen and further certified by the 16S rRNA sequencing. For species-level identification, 16S rRNA gene sequences were compared to those in the GenBank database using the BLAST tool (NCBI). Our data were aligned with a 16S rDNA sequence dataset for phylogenetic analysis using the BioEdit tool. The MEGA 7.0 program was used to construct the phylogenetic analysis. The strains were kept at – 80 °C in 20% (v/v) of glycerin solution. The subsequent fermentation test with the selected strains executed MCA and PA measurements.

### Measurement of MCA and PA

The fermentation broth was centrifuged at 5000 × g for 10 min. The crude MCE collected from the supernatant was utilized to measure MCA and PA according to the method of Arima et al. (1967) with some modifications. The 10% (w/v) skimmed milk substrate containing 10 mM CaCl_2_ was incubated at 35 °C for 10 min. Observing curd production by rotating the tube frequently until visible discrete particles appeared on its inner surface. The MCA is expressed in Soxhlet units (SU), which represents the amount of enzyme that can clot 1 mL of the milk substrate at 35 °C. The MCA was determined using the following formula: SU = (2400 × V_S_ × N)/(T × V_E_), where V_S_ is the milk volume (mL), N is the dilution of MCE, T is the milk-clotting time (s) and V_E_ is the MCE volume (mL). For the measurement of PA, the amount of MCE that releases 1 g of tyrosine per 1 mL in 1 min is defined as one unit (1 U).

### Strain growth and DB219 MCE production

The strain stored at − 80 °C was inoculated to the modified TYC medium and incubated at 37 °C for 12 h. Then, a single colony was inoculated into a 250 mL flask containing 50 mL of modified TYC medium and incubated at 37 °C for 10–12 h under shaking at 180 rpm as seed liquid. Three 250 mL of flasks (30 g/L wheat bran shorts) were autoclaved at 115 °C for 20 min and inoculated with 5% (v/v) inoculum size to produce MCE. The fermentation parameters were as follows: temperature 37 °C, agitation speed 180 rpm, initial pH 6.15 and volume 50 mL. Samples were taken every 12 h from 0 to 60 h and centrifuged for 10 min at 5000×*g* and 4 °C. The MCA and PA of MCE were determined using the fermentation supernatant.

### Optimization of fermentation conditions

#### The effects of wheat bran concentrations on DB219 MCE production

The wheat bran shorts concentration included 20, 30, 40, 50, 60 and 70 g/L. The initial pH was 6.15 and 5% of inoculum size was added to a 250 mL flask containing 50 mL wheat bran medium without extra carbon and nitrogen sources at 37 °C and 180 rpm. MCA was measured for 12–60 h every 12 h.

#### The effects of carbon and nitrogen sources on DB219 MCE production

The carbon sources were optimized through adding 10 g/L glucose, sucrose, soluble starch, maltodextrin and lactose to wheat bran medium. The MCA in the wheat bran medium (without extra carbon sources) was the control and taken as 100%. The optimal nitrogen sources were optimized through adding 3 g/L corn steep liquor, casein peptone, urea, yeast extract powder, ammonium sulfate and ammonium citrate to wheat bran medium containing the optimal carbon source. The MCA in the wheat bran medium containing 10 g/L optimal carbon source (without extra nitrogen sources) was the control and considered 100%. The concentration optimization of carbon and nitrogen sources was carried out based on optimal carbon source (2.5, 5, 10, 12.5, 15 and 20 g/L) and optimal nitrogen source (1, 2, 3, 4, 5 and 6 g/L). The 10 g/L optimal carbon source and 3 g/L optimal nitrogen source were the control. The MCA was measured every 12 h from 12 to 60 h.

#### The effects of bioprocess parameters on DB219 MCE production

The effect of inoculum size (1, 3, 5, 7, 9 and 11%, v/v) and fermentation medium volume (20, 30, 40, 50, 60 and 70 mL) on MCE production was evaluated. The initial pH of the fermentation medium was chose as 4.15, 5.15, 6.15, 7.15, 8.15 and 9.15. The fermentation medium contained the optimal carbon and nitrogen source. The fermentation process was conducted at 37 °C and 180 rpm. The MCA was measured every 12 h from 12 to 60 h.

#### Plackett–Burman design

Plackett–Burman design tested six factors on fermentation including wheat bran concentration, inoculum size, optimal carbon source concentration, optimal nitrogen source concentration, volume and initial pH. A single factor experiment determined the selection of twelve runs of factors (A-F) at two levels (+ 1 and –1). The results were fitted by Mini Tab 2.0.

#### Path of steepest ascent/descent

The Plackett–Burman design relevant factors over the 95% confidence level (*P* < 0.05) were chosen and optimized further using the steepest ascent/descent method. Experiments were conducted at predetermined intervals along the steepest ascent/descent. Three factors were selected for the steepest ascent/descent experiment: wheat bran concentration (A), optimal nitrogen source concentration (C) and volume (E).

#### Box-Behnken design

The DB219 MCE production was optimized through an RSM-based Box Behnken design model (BBD). Based on the above experiment finding, three factors wheat bran concentration (A), optimal nitrogen source concentration (B) and volume (C) were chosen and tuned at three levels to maximize the decolorization process. Analysis of Variance (ANOVA) was used to evaluate the viability of design model.

### Statistical analysis

Each experiment was conducted in triplicate. Means and standard deviations were used to express the findings. The acquired data were analyzed by SPSS 17.0, one-way ANOVA and Minitab 2.0. Box-Behnken design was executed by Design-Export V8.0.6. The statistical significance was established at *P* < 0.05.

## Results

### Screening and identification of MCE-producing strain

A total of 40 strains were isolated and purified from soil samples. On the solid casein media, 12 strains displayed significant or moderate precipitation rings and small hydrolysis rings, indicating high MCA/PA ratio (Additional file 1: Table S1). The analysis of strain milk-clotting characteristics and morphology demonstrated obviously different on MCE production capacity and characteristics of MCE. In order to observe and further compare the differences of these strains, 15 strains were spotted on casein plates. The colony zone diameter (CD), precipitation ring diameter (PD) and hydrolysis ring diameter (HD) were recorded (Table 1).

**Table 1:** The diameter of hydrolysis and precipitation rings on the solid casein medium

CN	CD (mm)	HD (mm)	PD (mm)	P/C	P/H
YN111	4.0 ± 1.0	5.0 ± 1.0	17.0 ± 2.0	4.3	3.4
DB219	3.0 ± 0.5	3.5 ± 0.5	19.0 ± 2.0	6.3	5.4
QD111	1.5 ± 0.5	5.5 ± 1.0	11.0 ± 1.5	7.3	2.0
DB215	4.0 ± 1.0	7.0 ± 1.0	11.5 ± 1.5	2.9	1.6
DB218	2.0 ± 0.5	3.0 ± 0.5	10.5 ± 1.5	5.3	3.5
YN213	4.0 ± 1.0	6.0 ± 1.0	14.0 ± 2.0	3.3	2.2
NX112	4.0 ± 0.5	8.0 ± 1.0	13.0 ± 1.0	3.3	1.6
GS113	2.0 ± 0.5	5.0 ± 1.0	10.0 ± 1.5	5.0	2.0
GL111	4.0 ± 1.0	5.0 ± 1.0	9.0 ± 1.0	2.3	1.8
DB215	4.0 ± 1.0	7.0 ± 1.0	11.5 ± 1.5	2.9	1.6
LY11	4.0 ± 1.0	4.0 ± 1.0	17.0 ± 2.0	4.3	4.2
QD212	3.0 ± 0.5	4.0 ± 1.0	16.5 ± 1.5	5.5	4.1

The data are represented as Mean ± SD (n = 3). CN: Strain number; CD: Colony zone diameter; HD: Hydrolysis ring diameter; PD: Precipitation ring diameter; P/C: The ratio of PD to CD; P/H: The ratio of PD to HD. The culture time of all strains was 24 h

Furthermore, 16S rRNA sequencing was used to investigate the strains. Among the 12 strains, the pathogenic bacteria (e.g., *Bacillus cereus*, *Acinetobacter* and *Bacillus wiedemann*) were eliminated. The other 6 nonpathogenic strains (DB219, GS113, QD212, DB215, LY11 and NX112) formed large precipitation rings in solid casein medium and were selected for the subsequent fermentation experiment (Data not shown). As shown in Table 2, the fermentation filtrates of DB219, GS113 and QD212 strains had MCA. Their MCA were 734.85, 40.04 and 377.98 SU/mL, respectively. The DB219 strain had the highest MCA compared with other strains. DB219 strain was selected for MCE production.

**Table 2:** The maximal MCA of strains in wheat bran fermentation medium

Strain number	Maximal MCA (SU/mL)
DB219	753.85 ± 13.12
GS113	40.04 ± 2.50
QD212	377.98 ± 4.21
YN111	1.71 ± 0.08
LY11	1.39 ± 0.62
NX112	1.80 ± 0.11

**Fig. 1:**
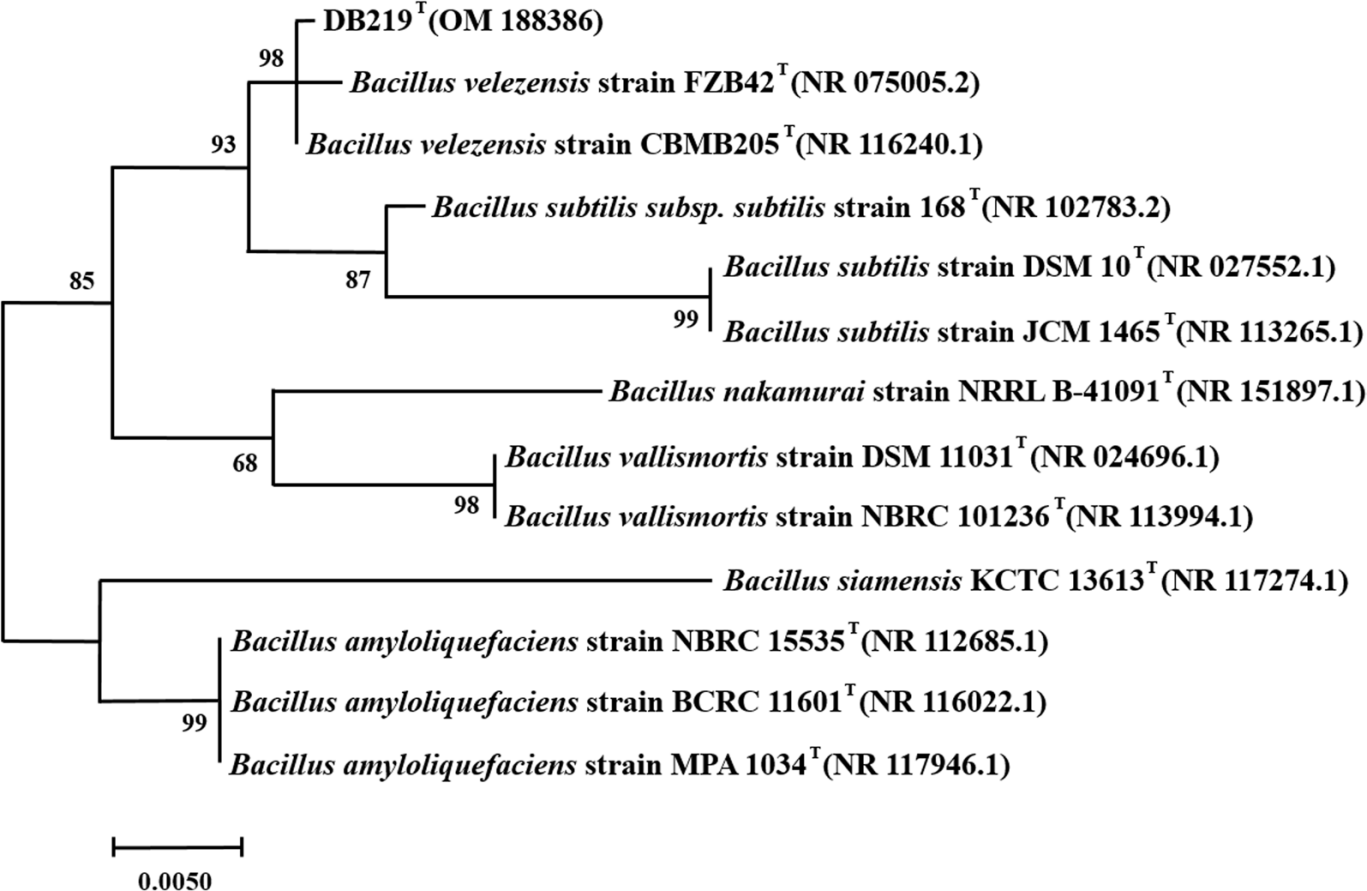
Phylogenetic tree based on 16S rRNA gene sequences of the isolated DB219. GenBank accession numbers are given in parentheses. Numbers at nodes refer to bootstrap values (based on 1000 replicates)

The physiological and biochemical characteristics of DB219 strain were shown in Additional file 1: Table S2. DB219 strain could be fermented oxidatively through glucose and starch. DB219 strain was unable to generate hydrogen sulfide gas. The V-P test demonstrated that the DB219 strain could hydrolyze glucose into pyruvate and decarboxylate pyruvate that was degraded into acetyl methyl methanol. The methyl red test revealed that sugar metabolism created minimal acid through strain or the acid is transformed into other molecules. However, DB219 strain could not utilize citric acid. Meanwhile, DB219 strain was found based on homology analysis of 16S rRNA sequences in GenBank for species identification. DB219 strain is more closely related (98% homology) to *Bacillus velezensis* strain FZB42 (NR075005.2) and *Bacillus velezensis* strain CBMB205 (NR116240.1) (Fig. 1). DB219 strain was identified as *Bacillus velezensis* through the above analyses. *Bacillus velezensis* DB219 (OM188386) was deposited in the China General Microbiological Culture Collection Center (CGMCC No. 24614).

### Growth and MCE-producing characteristics of *Bacillus velezensis* DB219

**Fig. 2:**
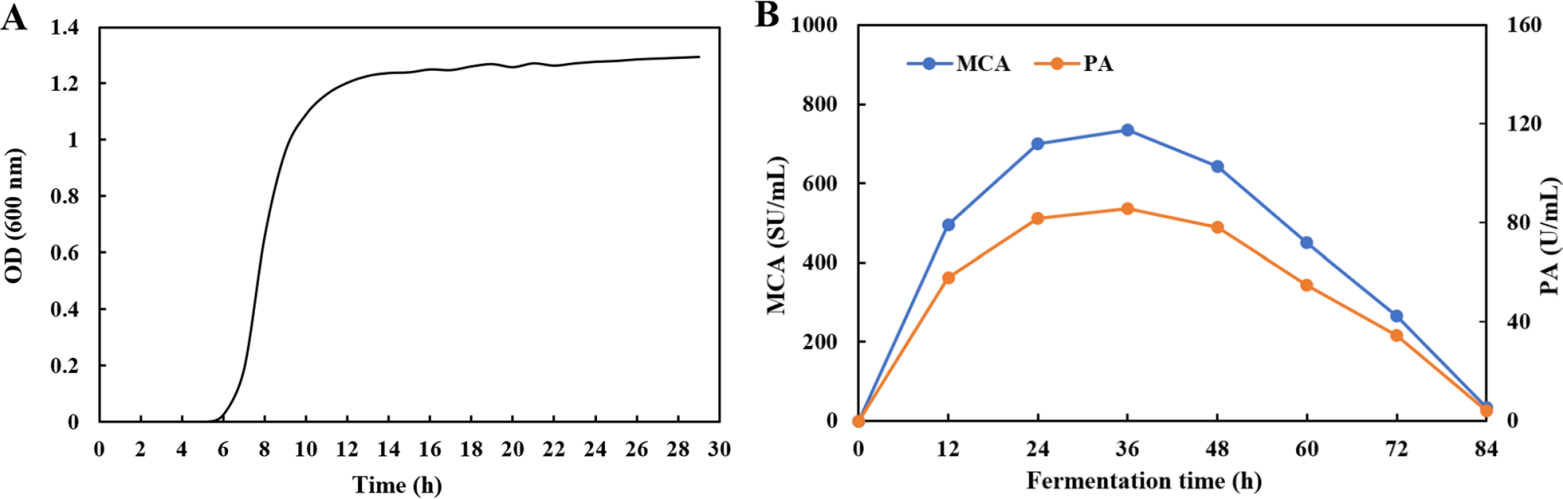
Growth of *Bacillus velezensis* DB219 in modified TYC medium within 30 h (**A**) and DB219 MCE production during different fermentation times (12–84 h) (**B**). The data are represented as mean ± SD (n = 3)

As shown in Fig. 2A, DB219 grew quickly in the modified TYC medium. Figure 2B showed the MCA and PA of DB219 MCE. DB219 MCE achieved the highest MCA (754 ± 13 SU/mL) and PA (82 ± 6 U/mL) at 36 h. The MCA/PA ratio was 9.2 throughout the fermentation.

### Optimization of fermentation conditions of *Bacillus velezensis* DB219

#### Wheat bran concentration tawards MCE production

**Fig. 3:**
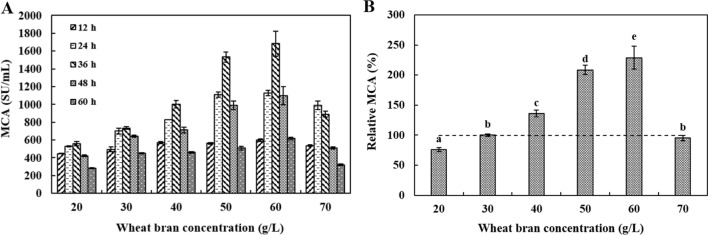
The effects of different wheat bran concentration on DB219 MCE production during fermentation (12–60 h). ^a−e^ Values in the same row with different superscripts are significantly different (*P* < 0.05). The data are represented as mean ± SD (n = 3)

Figure 3 indicated that MCA tended to increase and then decrease with the increase of wheat bran concentration. The MCA reached the maximum of 754 ± 13 SU/mL at wheat bran concentration of 60 g/L and 36 h, which was 8% higher than the control. Therfore, the 60 g/L wheat bran shorts was selected as the primary fermentation medium.

#### Kinds of carbon and nitrogen sources tawards MCE production

**Fig. 4:**
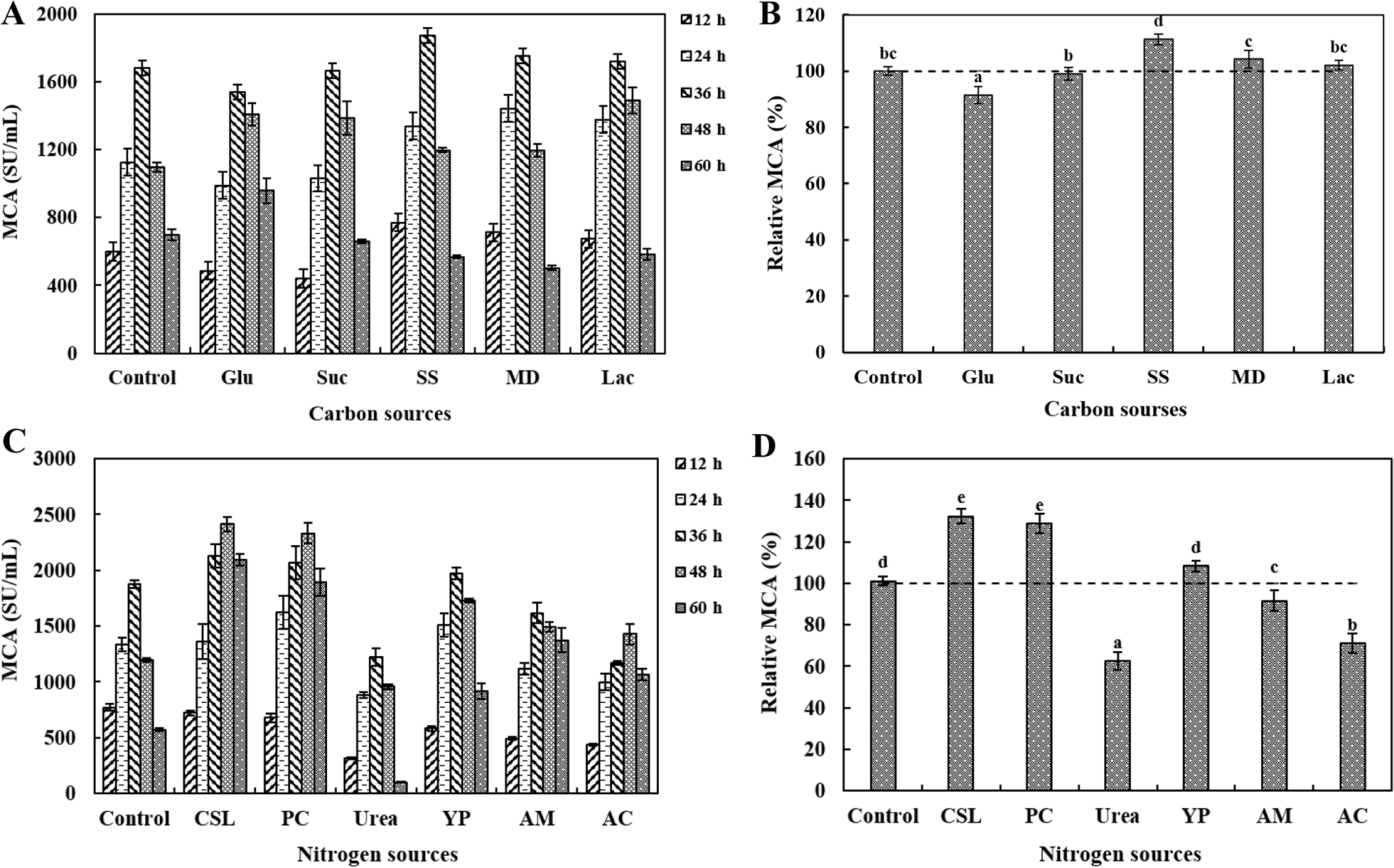
The effects of different kinds of carbon sources on DB219 MCE production during fermentation (12–60 h) (**A**, **B**). Glu, glucose; Suc, sucrose; SS, soluble starch; MD, Maltodextrin and Lac, Lactose. The effects of different kinds of nitrogen sources on DB219 MCE production during fermentation (12–60 h) (**C**, **D**). CSL, corn steep liquor; PC, casein peptone; YP, yeast extract powder; AM, ammonium sulfate and AC, ammonium citrate. ^a−e^ Values in the same row with different superscripts are significantly different (*P* < 0.05). The data are represented as mean ± SD (n = 3)

AS shown in Fig. 4A, B, the highest MCA was 1873 ± 36 SU/mL at 48 h when the 10 g/L soluble starch was used as carbon source, which was 11% higher than the control. Sucrose, maltodextrin and lactose have little effect on MCE production. Glucose inhibited the MCE production, which was 8% lower than the control. Figure 4C, D showed the effect of 3 g/L organic nitrogen sources (corn steep liquor, casein peptone, urea and yeast extract powder) and 3 g/L inorganic sources (ammonium sulfate and ammonium citrate) on MCE production. The organic nitrogen sources could significantly increase MCE production compared with inorganic nitrogen sources. The highest MCA (2331 ± 92 SU/mL) was achieved at presence of corn steep liquor. The corn steep liquor and casein peptone increased MCA by 32% and 28%, respectively. Urea, ammonium sulfate and ammonium citrate inhibited MCE production.

#### Inoculum size tawards MCE production

**Fig. 5:**
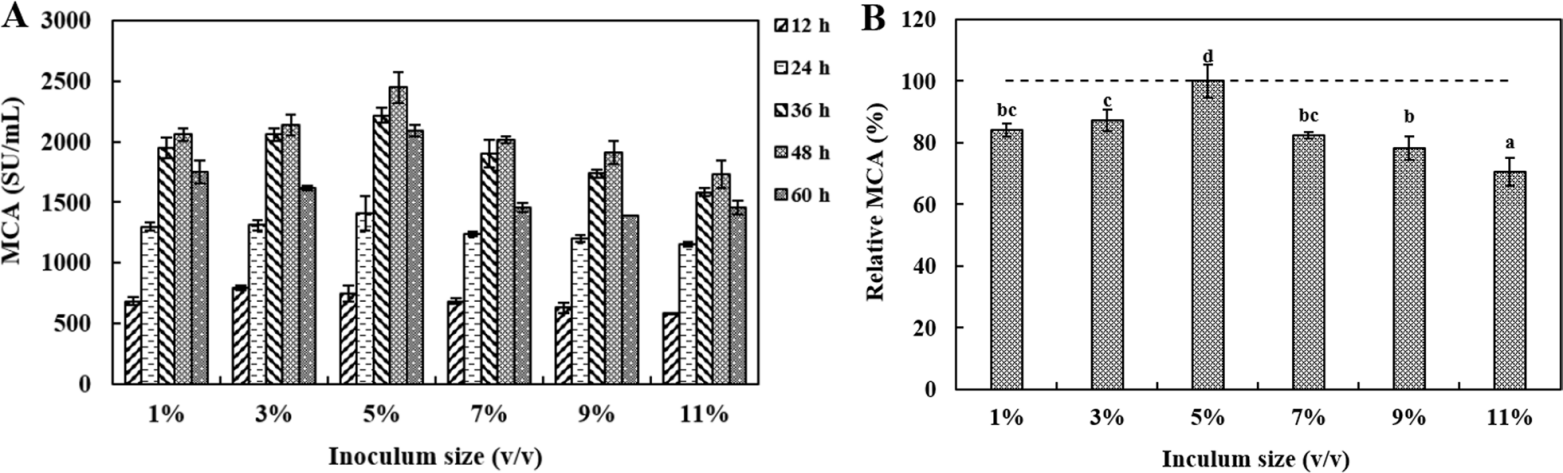
The effects of inoculum size on DB219 MCE production during different fermentation times (12–60 h). The medium composition was wheat bran (60 g/L), soluble starch (10 g/L) and corn steep liquor (3 g/L). ^a−d^ Values in the same row with different superscripts are significantly different (*P* < 0.05). The data are represented as mean ± SD (n = 3)

Figure 5 indicated the effect of various inoculum size on MCE production. It is observed that 5% (v/v) inoculum size led to maximal MCA (2445 ± 132 SU/mL) followed by 3% (v/v) and 7% (v/v) inoculum size.

#### Soluble starch and corn steep liquor concentration tawards MCE production

**Fig. 6:**
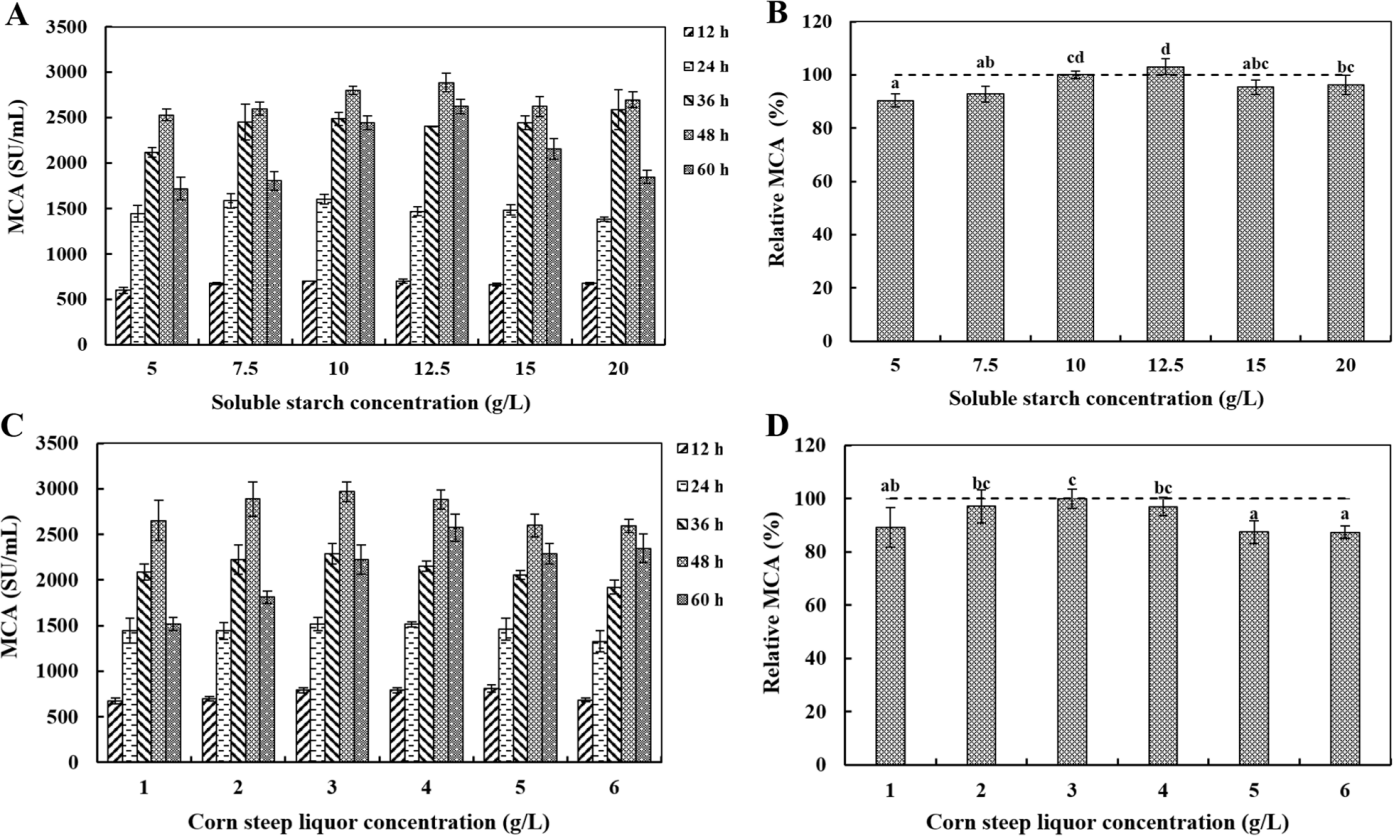
The effects of different soluble starch (optimal carbon source) concentration (**A**, **B**) and corn steep liquor (optimal nitrogen source) concentration (**C**, **D**) on DB219 MCE production during fermentation (12–60 h). ^a−d^ Values in the same row with different superscripts are significantly different (*P* < 0.05). Data were presented as means of triplicate measurements; Error bars were standard deviations. The data are represented as mean ± SD (n = 3)

As shown in Fig. 6A, B, the MCA increased and then decreased with increase of soluble starch in fermentation medium. The MCA was highest (2882.35 ± 101.89 SU/mL) when soluble starch concentration was 12.5 g/L. As indicated in Fig. 6C, D, the MCE production increased with increase of corn steep liquor concentration and reached the maximum (2971.65 ± 108.36 SU/mL) when fermentation medium contained 12.5 g/L soluble starch and 3 g/L corn steep liquor.

#### Initial pH and volume tawards MCE production

**Fig. 7:**
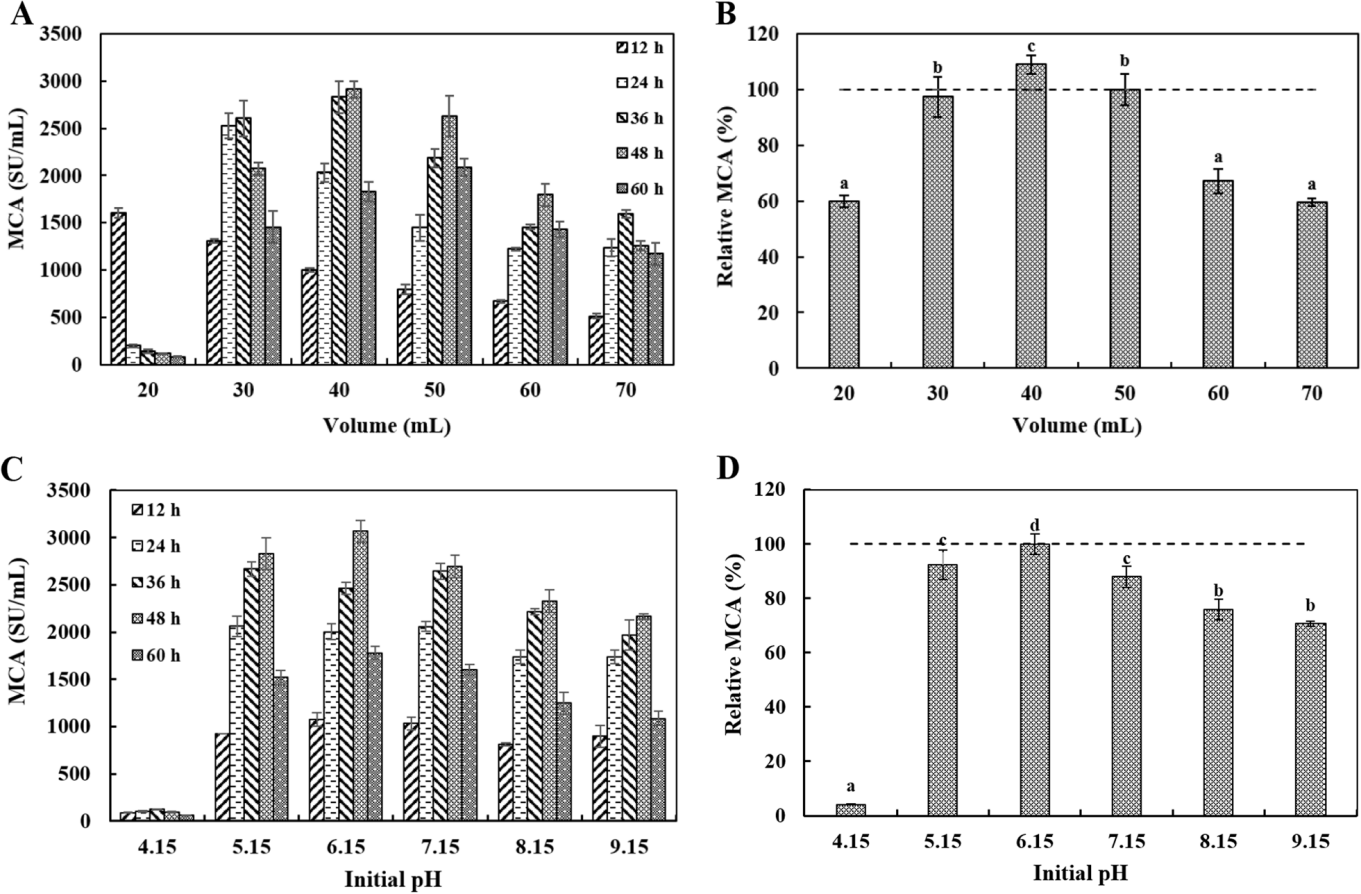
The effects of volume (A and B) and initial pH (C and D) of fermentation medium on DB219 MCE production during different fermentation times (12–60 h). The medium composition was wheat bran (60 g/L), soluble starch (12.5 g/L) and corn steep liquor (3 g/L). ^a−d^ Values in the same row with different superscripts are significantly different (*P* < 0.05). The data are represented as mean ± SD (n = 3)

Figure 7 showed the effect of initial pH and volume on MCE production. The MCE production reached the maximum at initial pH 6.15 and volume 40 mL in the 250 mL flask.

#### Plackett–Burman design and path of steepest ascent/descent

There are 6 variables in 13 Plackett–Burman design trials including A (wheat bran concentration), B (soluble starch concentration), C (corn steep liquor concentration), D (inoculum size), E (volume) and F (initial pH) as shown in Additional file 1: Table S3. ANOVA analysis of the experiment data for each response produced an F-value (12.96) and P-value (0.006) of this model. MCA exhibited significant response range from 1520.6 to 2485.4 SU/mL in 12 trials. P-values A, C and E were 0.013, 0.028, and 0.004, respectively. They were significant and had positive effect on MCE production. A linear regression model was constructed as follows: MCA = 1926.2 + 161.0 A + 51.2 B + 119.1 C – 68.3 D – 190.1 E – 40.1 F. The R^2^ value and adjusted R^2^ value were 0.9396 and 0.8670, respectively. Table 3 showed the steepest ascent/descent of wheat bran shorts concentration, corn steep liquor concentration and volume. The MCA increased from test 1 to test 5 and decreased at test 6. The maximal MCA (2965.55 SU/mL) was achieved at wheat bran concentration 60 g/L, corn steep liquor 3 g/L and volume 40 mL. It displayed a spot close to the maximal MCA zone. Further optimization was performed using the Box Behnken Design according to the near range of this point.

**Table 3:** The path of steepest ascent/descent experiments

Run	Wheat bran concentration (g/L)	Corn steep liquor concentration (g/L)	Volume (mL)	MCA (SU/mL)
1	50.0	2.00	50.0	2342.86
2	52.5	2.25	47.5	2433.88
3	55.0	2.50	45.0	2400.00
4	57.5	2.75	42.5	2823.53
5	60.0	3.00	40.0	2954.55
6	62.5	3.25	37.5	2745.10
7	65.0	3.50	35.0	2668.73
8	67.5	3.75	32.5	2560.46
9	70.0	4.00	30.0	2370.73

#### Box-Behnken design and validation

**Fig. 8:**
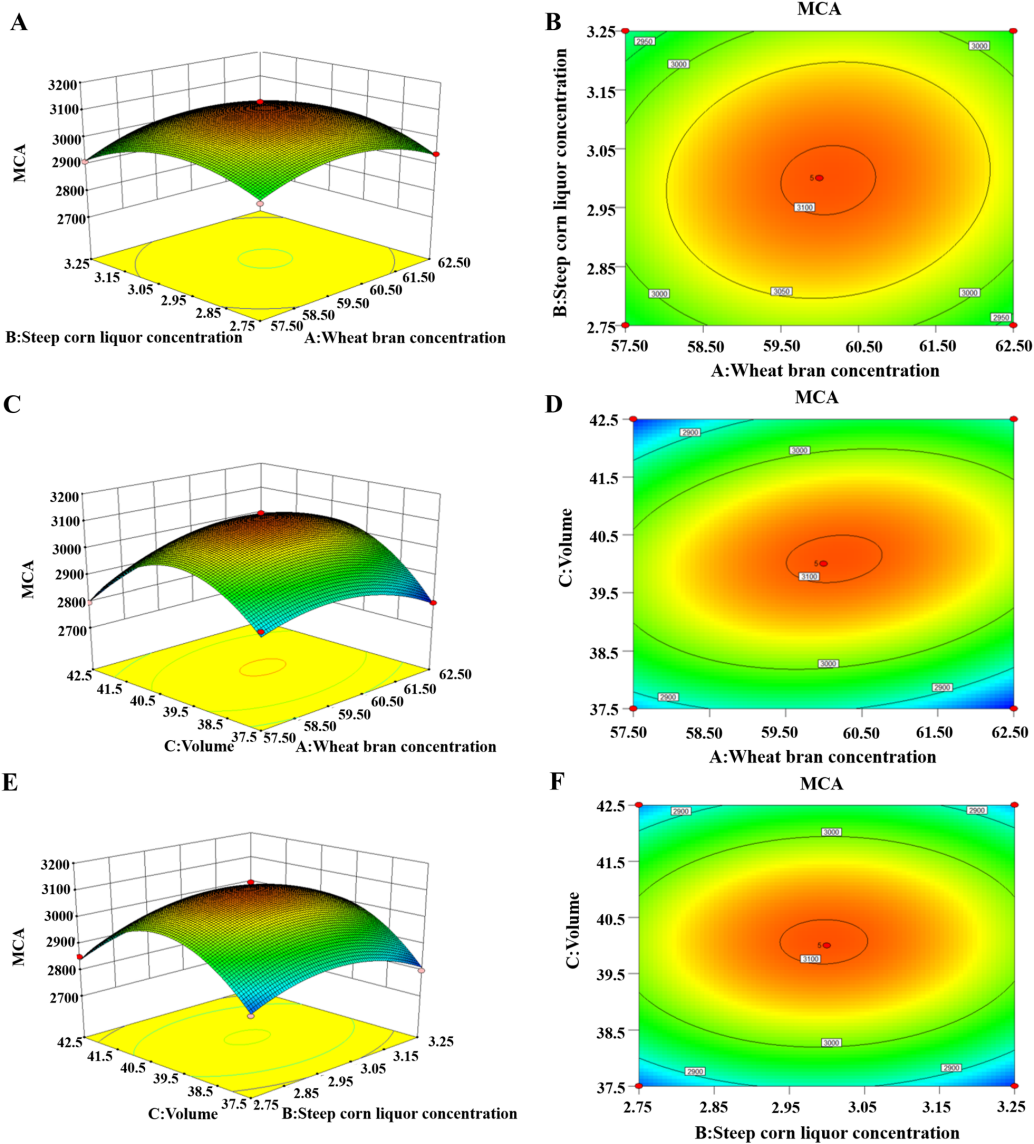
Response surface and contour map of wheat bran concentration and corn steep liquor concentration interaction (**A**, **B**), wheat bran concentration and volume interaction (**C**, **D**) and corn steep liquor concentration and volume interaction (**E**, **F**)

Additional file 1: Table S4 displayed the ANOVA findings for the quadratic response surface regression model. The F-value for the regression model was 40.29 and P-value was 0.0001. However, the lack of fit P-value was more than 0.5. The adjusted R^2^ was 0.9567, which was close to the R^2^ value. According to the F-value, the factors affected MCE production were C (volume) > A (wheat bran concentration) > B (corn steep liquor concentration). AB, A^2^, B^2^ and C^2^ were the most significant characteristics (*P* < 0.05). The regression model followed mathematical statistic principles and could be used to predict DB219 MCE production. Regression equation was generated according to standard ANOVA. MCA =  + 3104.52 + 7.35 A – 2.93 B + 10.29 * C + 14.71 * A * B + 41.97 * AC + 7.13 * B * C – 79.18 * A^2^ – 85.50 * B^2^ – 186.73 * C^2^. As shown in Fig. 8, the maximal expected MCA was 3104.49 SU/mL at wheat bran concentration 60.14 g/L, corn steep liquor concentration 3 g/L and volume 40.08 mL through the quadratic response surface regression model and software analysis. The model validation was carried out by performing the experiments at predicted fermentation conditions. Experimental value for maximal MCA was 3164.84 SU/mL, which was 101.9% of the predicted value.

## Discussion

A strain of *Bacillus velezensis* DB219 was isolated and identified from Wuchang “black soil”. *Bacillus velezensis* DB219 showed high MCE production. DB219 MCE exhibited high P/C, P/H and MCA/PA ratio (9.2), which made it appropriate for cheese production. The cheese made with MCE with greater MCA (big precipitation ring) and lower PA (small hydrolysis ring) might result in less bitter peptide production (Bouazizi et al. 2022). P/C ratio determines the MCE production capacity of the strains. The larger the P/C ratio, the greater the MCE production of strain was. The P/H ratio is one of the important markers for detecting MCE applicability. The greater the P/H ratio, the higher MCA/PA ratio was (Meng et al. 2018). Therefore, the *Bacillus velezensis* DB219 with the high P/C and P/H ratio was suitable for cheese making. Cheese contained functional peptides generated from the casein by MCE (Mane et al. 2019). However, excessive hydrolysis could produce much bitter peptides, which can affect the taste and flavor of cheese. Therefore, the MCA/PA ratio is a crucial factor to assess MCE in cheese making. It is comparable to the MCA/PA ratio of the MCE screened by Lizardi-Jiménez et al. (2019) from *Rhizopus microspores var. chinensis*. It is similar to that of the widely used commercial MCE (e.g., calf rennet and *R. miehei* MCE) (Thakur et al. 1990). Therefore, the *Bacillus velezensis* DB219 MCE with high MCA/PA ratio (9.2) was suitable for cheese making and could be considered as an appropriate traditional substitute.

*Bacillus velezensis* DB219 grew fast in the modified TYC medium (Fig. 2A), indicating that MCE production occurred soon. It could save expenses and increase equipment usage during MCE production. Wheat bran was an affordable energy source and agro-industrial byproduct and had great potential for enzyme fermentation production. Wheat bran provided about 15% protein and other essential nutrients (e.g., cellulose, starch and trace elements) for microbial growth and enzyme synthesis (Zhang et al. 2013). Wheat bran was involved in a wide range of enzyme production as a substrate for fermentation (Ding et al. 2011). The nutrients were inadequate when the substrate concentration was low. However, when the wheat bran concentration is high, the dissolved oxygen in the water was low and not favorable to the solubility of nutrients, which is not conducive to the culture of strains and the creation of metabolites (Liu et al. 2021). Moreover, the cheap and effectively used carbon and nitrogen sources were the primary factors for commercial production of MCE (Patel et al. 2005). Different strains perferred vrious carbon and nitrogen sources. Soluble starch and corn steep liquor were the optimal carbon source and nitrogen source for DB219 MCE production (Fig. 4). The optimal carbon source for most *Bacillus* spp. to produce MCE was glucose (Cheng et al. 2008; Shieh et al. 2009). The highest yields were obtained when *Bacillus cereus* produced keratinase with the addition of 1% lactose and casein as carbon and nitrogen sources (Arokiyaraj et al. 2019). Glucose as a quick-acting carbon source was convenient for microorganism utilization. Therefore, the addition of glucose caused the strain growing too fast so that the nutrient was insufficient for subsequent MCE production (Naveed et al. 2022). Soluble starch was a polysaccharide and called as “slow-acting carbon source”. It could provide long-lasting energy for strain growth and enzyme production (Ruiz et al. 2010). In addition, the organic nitrogen source was more beneficial to DB219 MCE production than inorganic nitrogen source (Fig. 4C, D). DB219 synthesized inorganic nitrogen into amino acids, which decreased the growth of microorganisms. However, organic nitrogen contains many amino acids that can be directly absorbed by DB219 (Sathya et al. 2009). DB219 MCE production decreased gradually with the increase of corn steep liquor. MCE synthesis was inhibited when the concentration of carbon and nitrogen source were excessive. Therefore, it is necessary to determine the optimal carbon and nitrogen concentration for increasing MCE production and reducing fermentation cost.

The optimal inoculum size was worthy of study. A higher optimal inoculum size affected the availability of the substrate and a lower optimal inoculum size caused longer fermentation time (Shah et al. 2021). Unsuitable inoculum size could have a significant effect on fermentation efficiency. It is possible that the MCA is reduced due to the insufficient nutrients caused by the fast growth of strains and large biomass due to excessive inoculation. The optimal inoculum size for *Bacillus velezensis* DB219 to produce MCE was 4%, which was different from that for *Bacillus amyloliquefaciens* D4 (4%) (He et al. 2012). The initial pH of medium significantly affected the bacterial membrane charge, membrane permeability and nutrient ionization, thereby affecting the absorption of nutrient through various membrane transport and enzymatic activities (Moon et al. 2010). “Optimal pH” referred to the H^+^ and OH^–^ ions had the greatest effect on ionic and hydrogen bonds within the enzyme at this pH, which made the active site of enzyme complementary to the substrate (Limkar et al. 2019). At pH 4.15, MCA almost disappeared completely. It might be denaturation or inactivation of the MCE (Bergamasco et al. 2000). The pH 6.15 is the best initial medium pH for DB219 MCE production, which saved time and cost. However, the optimal initial pH value of *Bacillus velezensis* ASN1 for cellulase production was 4.7 (Nair et al. 2018). The optimal pH for prolyl aminopeptidase by *Bacillus subtilis* was 7.0, which was different from *Bacillus velezensis* DB219 (Wang et al. 2018). In addition, the volume of liquid mainly affected the dissolved oxygen. The less volume, the higher the dissolved oxygen was. However, it also increased the evaporation of the solution during fermentation. Therefore, an appropriate volume of liquid should be found. When the volume was 20 mL, the DB219 MCE-producing time was significantly advanced due to the high dissolved oxygen resulting in vigorous strain growth and fast consumption of nutrient, which led to subsequent malnutrition and decreased MCE production. In contrast, when liquid medium was excessive, dissolved oxygen was not enough leading to slow strain growth and decreased MCE production (Limkar et al. 2019).

The F-value and P-value of regression model were 40.29 and 0.0001, indicating that the model was significant. However, the lack of fit P-value was more than 0.5, indicating that it was not significant compared to the pure error and the established model had a certain reference value. The adjusted R^2^ (0.9567) was close to the R^2^ value, indicating that the model could explain 95.67% of the finding. Regression equation was generated, indicating that MCA was independent factor and second polynomial equation. DB219 MCE achieved the maximal MCA (3164.84 SU/mL) and 4.3-fold higher than the control through response surface analysis. It demonstrated *Bacillus velezensis* DB219 was able to produce lots of and high quality of MCE in short time and cheap cost. *Bacillus velezensis* DB219 was potential as MCE-producing strain and applied to dairy production (e.g., cheese, fermented milk, ice cream and tofu). It is crucial to purify DB219 MCE and investigate milk-clotting characteristics in the future.

## Supplementary Information

**Additional file 1: Table S1.** Morphology and milk-clotting potential of strains in initial screening.** Table S2** Physiological and biochemical characteristics of Bacillus velezensis DB219.** Table S3.** Analysis of variance and regression analysis of Plackett-Burman design on MCE-producing optimization for Bacillus velezensis DB219.** Table S4** Analysis of variance and regression analysis of Box-Behnken design on MCE-producing optimization for Bacillus velezensis DB219.

## Data Availability

The datasets used and/or analyzed during the current study are available from the corresponding author on reasonable request.
