# Supplementary material for: Isolation and production optimization of a novel milk-clotting enzyme Bacillus velezensis DB219
Source: AMB Express. 2022 Nov 26;12:149. doi: 10.1186/s13568-022-01493-9 (PMC9701308; doi:10.1186/s13568-022-01493-9)
Supplement: Supplementary file 1 — Additional file 1: Table S1. Morphology and milk-clotting potential of strains in initial screening. Table S2 Physiological and biochemical characteristics of Bacillus velezensis DB219. Table S3. Analysis of variance and regression analysis of Plackett-Burman design on MCE-producing optimization for Bacillus velezensis DB219. Table S4 Analysis of variance and regression analysis of Box-Behnken design on MCE-producing optimization for Bacillus velezensis DB219. [file 13568_2022_1493_MOESM1_ESM.docx]

**Table S1** Morphology and milk-clotting potential of strains in initial screening

| Strain number | Morphology |
| --- | --- |
| YN111 | large precipitation ring, fast growing and slimy with neat edges |
| DB219 | large precipitation ring, rough and hyaline spore |
| QD111 | little hydrolysis ring, dirty white, opacification and dry shape |
| DB215 | little hydrolysis ring, bright white and slimy with neat edges |
| DB218 | large precipitation ring, large strain, thin and convex |
| YN213 | large precipitation ring, faint yellow and smooth |
| NX112 | large precipitation ring, spore and full colony |
| GS113 | little hydrolysis ring, spore and slimy |
| GL111 | little hydrolysis ring, dirty white and rough edges |
| DB215 | little hydrolysis ring, moderate precipitation ring and smooth |
| LY11 | large precipitation ring, fast growing and neat edges |
| QD212 | large precipitation ring, large strain and slimy |

YN111, *Bacillus thuringiensis*; DB219, *Bacillus velezensis*; QD111, *Acinetobacter*; DB215, *Bacillus cereus*; DB218, *Bacillus wiedemann*; YN213, *Bacillus cereus*; NX112, *Bacillus tokyoides*; GS113, *Bacillus altitudinis*; GL111, *Bacillus cereus*; DB215, *Bacillus cereus*; LY11, *Bacillus thuringiensis*; QD212, *Bacillus megaterium*.

**Table S2** Physiological and biochemical characteristics of *Bacillus velezensis* DB219

| Test Projects | Identification results |
| --- | --- |
| Starch hydrolysis | + |
| Voges-Proskauer reaction (VP) | + |
| Methyl red reaction (MR) | – |
| Hydrogen sulfide gas production | – |
| Citrate utilization | – |
| Glucose oxidative fermentation | + |
| Catalase reaction | + |

**Table S3** Analysis of variance and regression analysis of Plackett-Burman design on MCE-producing optimization for *Bacillus velezensis* DB219

| Run | Factors | | | | | | Response | Regression data | | | | |
| --- | --- | --- | --- | --- | --- | --- | --- | --- | --- | --- | --- | --- |
|  | A (g/L) | B(g/L) | C (g/L) | D (%) | E (mL) | F | MCA (SU/mL) | Term | Effects | F-value | P-value | Ranking |
| 1 | 50 (–1) | 7.5 (–1) | 1 (–1) | 7 (+1) | 50 (+1) | 7.15 (+1) | 1354.9 | A | 322.1 | 23.67 | 0.005^c^ | 2 |
| 2 | 60 (+1) | 12.5 (+1) | 3 (+1) | 5 (–1) | 50 (+1) | 7.15 (+1) | 2002.3 | B | 102.4 | 2.39 | 0.183 | 5 |
| 3 | 50 (–1) | 12.5 (+1) | 3 (+1) | 5 (–1) | 50 (+1) | 6.15 (–1) | 1973.9 | C | 238.2 | 12.95 | 0.016^c^ | 3 |
| 4 | 50 (–1) | 7.5 (–1) | 1 (–1) | 5 (–1) | 40 (–1) | 6.15 (–1) | 1835.4 | D | -136.7 | 4.26 | 0.094 | 4 |
| 5 | 60 (+1) | 7.5 (–1) | 1 (–1) | 5 (–1) | 50 (+1) | 7.15 (+1) | 1834.5 | E | -380.2 | 32.99 | 0.002^c^ | 1 |
| 6 | 60 (+1) | 7.5 (–1) | 3 (+1) | 7 (+1) | 40 (–1) | 7.15 (+1) | 2218.9 | F | -82.2 | 1.47 | 0.280 | 6 |
| 7 | 50 (–1) | 12.5 (+1) | 3 (+1) | 7 (+1) | 40 (–1) | 7.15 (+1) | 2070.4 | Analysis of variance | | | | |
| 8 | 50 (–1) | 12.5 (+1) | 1 (–1) | 5 (–1) | 40 (–1) | 7.15 (+1) | 1835.4 |  |  |  |  |  |
| 9 | 50 (–1) | 7.5 (–1) | 3 (+1) | 7 (+1) | 50 (+1) | 6.15 (–1) | 1520.6 | Sourse | df | Mean square | F-value | P-Value |
| 10 | 60 (+1) | 12.5 (+1) | 1 (–1) | 7 (+1) | 50 (+1) | 6.15 (–1) | 1730.1 | Model | 6 | 170305 | 12.96 | 0.006 |
| 11 | 60 (+1) | 7.5 (–1) | 3 (+1) | 5 (–1) | 40 (–1) | 6.15 (–1) | 2485.4 | Residual | 5 | 13145 |  |  |
| 12 | 60 (+1) | 12.5 (+1) | 1 (–1) | 7 (+1) | 40 (–1) | 6.15 (–1) | 2252.0 | Cor Total | 11 |  |  |  |

Results were obtained using Minitab 2.0 software. A, wheat bran concentration; B, carbon source concentration; C, nitrogen source concentration; D, inoculum size; E, volume; F, initial pH and Y, MCA. Significant at *P* < 0.05.

**Table S4** Analysis of variance and regression analysis of Box-Behnken design on MCE-producing optimization for *Bacillus velezensis* DB219

| Run | BBD experiments | | | | ANOVA | | | | | | | Significance |
| --- | --- | --- | --- | --- | --- | --- | --- | --- | --- | --- | --- | --- |
|  | A^b^ | B | C | MCA (SU/mL) | Source | Sum of Squares | df | Mean Square | F-value | | P-value |  |
| 1 | 57.50 (–1) | 3.00 (0) | 42.50 (+1) | 2796.64 | Model | 2.323E+005 | 9 | 25811.91 | 40.29 | | < 0.0001 | ** |
| 2 | 62.50 (+1) | 3.00 (0) | 42.50 (+1) | 2880.57 | A | 432.53 | 1 | 432.53 | 0.68 | | 0.4383 |  |
| 3 | 60.00 (0) | 2.75 (–1) | 42.50 (+1) | 2852.05 | B | 68.90 | 1 | 68.90 | 0.11 | | 0.7525 |  |
| 4 | 60.00 (0) | 3.00 (0) | 40.00 (0) | 3131.18 | C | 846.70 | 1 | 846.70 | 1.32 | | 0.2880 |  |
| 5 | 62.50 (+1) | 3.00 (0) | 37.50 (–1) | 2796.64 | AB | 865.05 | 1 | 865.05 | 1.35 | | 0.2833 |  |
| 6 | 60.00 (0) | 3.25 (+1) | 42.50 (+1) | 2853.68 | AC | 7044.54 | 1 | 7044.54 | 11.00 | | 0.0128 | * |
| 7 | 62.50 (+1) | 2.75 (–1) | 40.00 (0) | 2939.39 | BC | 203.35 | 1 | 203.35 | 0.32 | | 0.5907 |  |
| 8 | 60.00 (0) | 2.75 (–1) | 37.50 (–1) | 2825.16 | A^2^ | 26398.21 | 1 | 26398.21 | 41.21 | | 0.0004 | ** |
| 9 | 60.00 (0) | 3.25 (+1) | 37.50 (–1) | 2798.27 | B^2^ | 30777.05 | 1 | 30777.05 | 48.05 | | 0.0002 | ** |
| 10 | 57.50 (–1) | 3.00 (0) | 37.50 (–1) | 2880.57 | C^2^ | 1.468E+005 | 1 | 1.468E+005 | 229.19 | | < 0.0001 | ** |
| 11 | 60.00 (0) | 3.00 (0) | 40.00 (0) | 3064.52 | Lack of Fit | 1370.65 | 3 | 456.88 | 0.59 | | 0.6550 |  |
| 12 | 60.00 (0) | 3.00 (0) | 40.00 (0) | 3096.77 | Pure Error | 3113.42 | 4 | 778.36 |  | |  |  |
| 13 | 60.00 (0) | 3.00 (0) | 40.00 (0) | 3098.92 | Cor Total | 2.368E+005 | 16 | 25811.91 | 40.29 | | < 0.0001 | ** |
| 14 | 62.50 (+1) | 3.25 (+1) | 40.00 (0) | 2969.7 |  | | | | | | | |
| 15 | 60.00 (0) | 3.00 (0) | 40.00 (0) | 3131.18 | Credibility analysis of the regression equations | | | | | | | |
| 16 | 57.50 (–1) | 3.25 (+1) | 40.00 (0) | 2910.87 | Std. Dev. | 25.31 | | R-Squared | | 0.9811 | | |
| 17 | 57.50 (–1) | 2.75 (–1) | 40.00 (0) | 2939.39 | Mean | 2939.15 | | Adj R-Squared | | 0.9567 | | |
| Second-order polynomial equation | | | | | C.V. % | 0.86 | | Pred R-Squared | | 0.8868 | | |
| MCA = + 3104.52 + 7.35 A - 2.93 B + 10.29 * C + 14.71 * A * B + 41.97 * AC + 7.13 * B * C - 79.18 * A^2^ - 85.50 * B^2^ - 186.73 * C^2^ | | | | | PRESS | 26795.09 | | Adeq Precision | | 16.012 | | |

Results were obtained using Design-Export V8.0.6 software. A, wheat bran concentration (g/L); B, steep corn liquor concentration (g/L); C, volume (mL).
